# Blue Shifted Carbon Dots-Based Fluorescent Probe for Determination of Ticagrelor: A Dual Method Evaluation Via AGREE and BAGI

**DOI:** 10.1007/s10895-025-04618-y

**Published:** 2025-11-17

**Authors:** Abdelrahman M. Allam, Azza Aziz M. Moustafa, Shereen A. Boltia, Sally S. El-Mosallamy

**Affiliations:** Pharmaceutical Analytical Chemistry Department, Faculty of Pharmacy, Cairo University, Kasr El-Aini St, P.O. Box 11562, Cairo, Egypt

**Keywords:** Blue shifted carbon quantum dots, Fluorescence quenching, Inner filter effect, Ticagrelor, AGREE, BAGI

## Abstract

This research presents the development of an innovative, environmentally sustainable, economical fluorimetric method for the detection of Ticagrelor (TICA) using oxygen rich carbon quantum dots (O-CQDs) prepared via a green pyrolysis technique from gallic acid, urea, and sodium edetate. The resulting O-CQDs exhibited a blue shifted maximum excitation (λ_ex_/λ_em_ of 290/417 nm) high quantum yield of 27.85 ± 0.89%, excellent photostability, and strong fluorescence emission, making them ideal candidates for analytical applications. Characterization of the synthesized O-CQDs was conducted with different techniques; high resolution transmission electron microscopy (HRTEM) coupled with energy-dispersive X-ray spectroscopy (EDX), Dynamic light scattering (DLS), and Fourier-transform infrared spectroscopy (FTIR). The fluorescent probe showed a linear response for TICA over a concentration range of 0.50–16.00 µg/mL, and LOD of 0.21 µg/mL. The quenching mechanism was investigated and attributed primarily to the inner filter effect (IFE) and dynamic quenching. The method was successfully applied to commercial pill formulations with high accuracy and precision. Additionally, the greenness and applicability of the proposed method were successfully examined using the AGREE and BAGI tools, proving the method’s consistency with green chemistry concepts and real-world applicability.

**Graphical Abstract:**

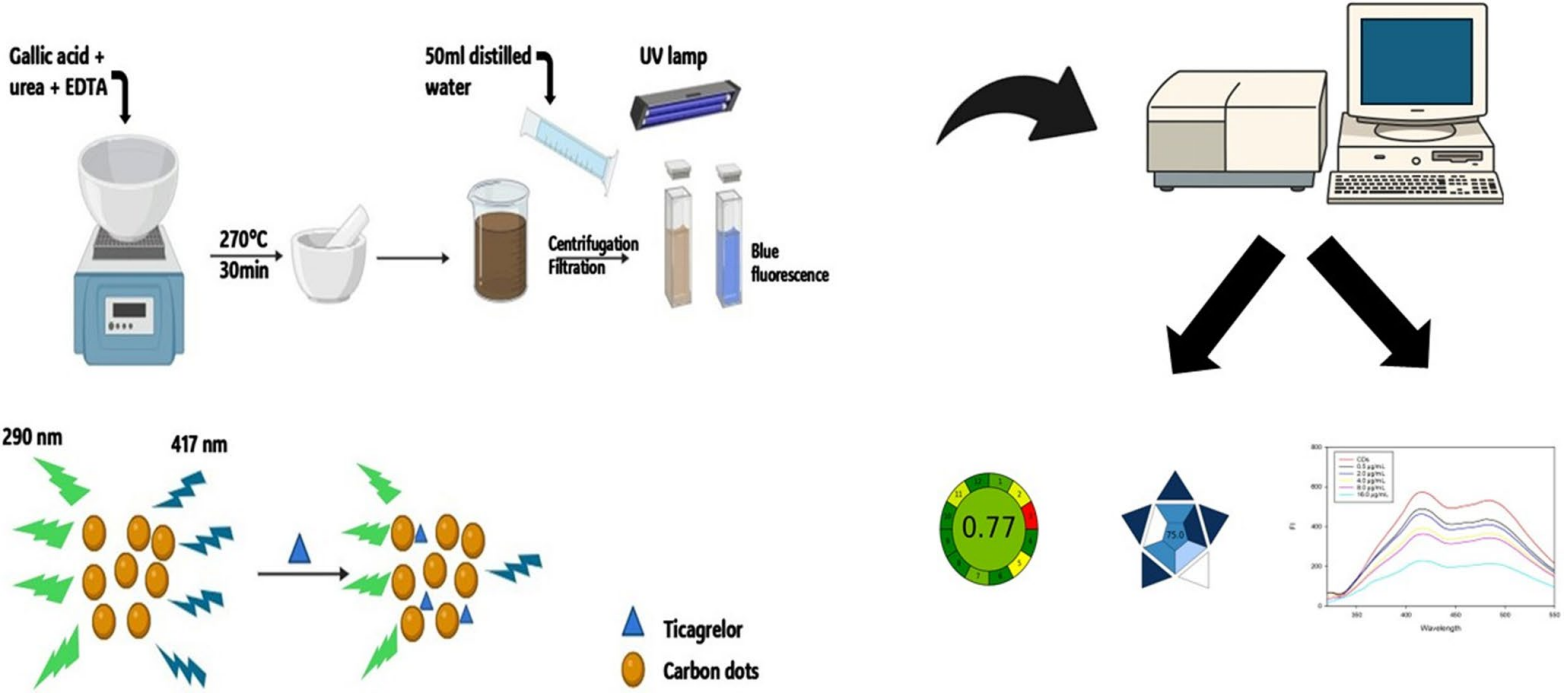

**Supplementary Information:**

The online version contains supplementary material available at 10.1007/s10895-025-04618-y.

## Introduction

Carbon quantum dots (CQDs) are a distinct category of carbon-based nanomaterials that have attracted much interest owing to their remarkable physicochemical and optical properties. These nanoparticles are generally less than 10 nm [1, 2], and provide numerous advantages compared to traditional fluorescent materials. Their principal characteristics include high water dispersibility, chemical stability, surface functionalization, robust photostability, and little cytotoxicity. These attributes place CQDs as prospective candidates across multiple disciplines, such as biomedical imaging and pharmaceutical delivery, catalysis, and environmental sensing. Their intrinsic biocompatibility and minimal toxicity facilitate secure application in biological applications. These characteristics have stimulated comprehensive research into the creation of CQD-based fluorescence sensors for both quantitative and qualitative analysis in pharmaceutical and therapeutic settings [3, 4].

Carbon quantum dots preparation strategies can be widely categorized into top-down and bottom-up approaches. The top-down approach entails the breakdown of carbon bulk materials using chemical oxidation, laser ablation, or electrochemical processing. Conversely, the bottom-up strategy forms CQDs from small molecule precursors employing methods such as thermal breakdown, hydrothermal approach, and microwave-assisted synthesis [5–8]. The latter approach is especially preferred for its simplicity, scalability, and alignment with green chemistry concepts. Carbon quantum dots are often doped with heteroatoms like nitrogen, sulfur, or phosphorus to improve their functional performance. Heteroatom doping greatly affects the photoluminescent properties, and surface reactivity of CQDs [9–11].

The synthesis approach, choice of dopants, and precursor materials collectively influence the optical and structural properties of CQDs, offering a versatile platform for tailoring nanoprobes for specific analytical applications. Although heteroatom doping generally enhances fluorescence intensity and enables tunable emission, it often results in a red shift of the excitation wavelength [12–15]. This shift may be undesirable when targeting non-fluorescent drugs, such as ticagrelor (TICA) (λ_max_ = 290 nm) -the drug under study-. These drugs absorb strongly in the mid-ultraviolet region, which can interfere with quenching based on the inner filter effect (IFE) Literature reports indicate that CQDs synthesized using oxygen-rich precursors tend to exhibit a blue shift in their excitation maxima compared to conventionally doped CQDs [2]. This optical behavior makes them more compatible with analytes absorbing in the mid-ultraviolet range. So, the challenge in this work is to meticulously choose bio-inspired oxygen doping agent like gallic acid (GA), along with disodium edetate (EDTA) and urea. As EDTA and gallic acid contain plenty of oxygen rich functional groups and urea contains few nitrogen containing functional groups, which enhance the resulting quantum yield without causing red shift to the λ_ex_ of the resulted oxygen enriched carbon quantum dots (O-CQDs).

Ticagrelor is an oral antiplatelet agent used to prevent atherothrombotic events in patients with acute coronary syndromes. It acts directly as a reversible P_2_Y_12_ receptor blocker, inhibiting platelet activation through a noncompetitive mechanism. The reported analytical techniques for TICA determination primarily rely on chromatographic methods [16–19], beside some spectrophotometric methods [20–23], voltametric methods [24–26], and only one spectrofluorimetric method [27]. However, to date, no analytical method has been reported utilizing CQDs for the fluorimetric determination of TICA.

The aim of the present study is to develop O-CQDs to be applied as a fluorescent based probe for the determination of TICA in both bulk powder and pharmaceutical formulation. To achieve this, a novel, simple, cost-effective, and green synthesis route was employed, involving the pyrolysis of bio-inspired substrate GA, along with urea, and EDTA. The prepared O-CQDs were comprehensively characterized to verify their structural and morphological features using high resolution transmission electron microscopy (HRTEM) coupled with energy-dispersive X-ray spectroscopy (EDX), Dynamic light scattering (DLS), and Fourier-transform infrared spectroscopy (FTIR).

## Experimental

### Materials and Reagents

Ticagrelor, certified to be 99.9% pure, was supplied by AstraZeneca Pharmaceutical Co. (Egypt). All chemicals used in the study including methanol (MeOH), gallic acid (GA), urea, disodium edetate (EDTA), quinine sulfate, acetic acid (HAc), boric acid (H_3_BO_3_), phosphoric acid (H_3_PO_4_), sodium hydroxide (NaOH), sulfuric acid (H_2_SO_4_), and sodium chloride (NaCl) were sourced from Sigma-Aldrich (Darmstadt, Germany). Brilique^®^ pills (90 mg, AstraZeneca) were procured from a local pharmacy. All reagents are of analytical grade.

Britton–Robinson buffer (BRB) with a pH range of 2.00 to 12.00 was prepared by mixing equal volumes of 0.04 mol L⁻¹ solutions of HAc, H_3_PO_4_, and H_3_BO_3_. The pH was subsequently modified to the desired value using 0.20 mol L⁻¹ NaOH.

### Instruments

Fluorescence measurements were conducted at ambient temperature utilizing a Jasco FP-6600 spectrofluorometer (Tokyo, Japan), fitted with a 1.00 cm quartz cuvette, employing excitation and emission slit widths of 5 and 10 nm, respectively. HRTEM was performed using a JEOL JEM-2100 electron microscope (JEOL Ltd., Tokyo, Japan), and elemental composition was confirmed by EDX (Thermo Fisher Scientific SEM-QUANTA FEG 250). Zeta potential and polydispersity index measurements were performed using DLS technique on a Malvern Zetasizer (Malvern Panalytical Ltd., Worcestershire, UK) at 25 °C. Fourier transform infrared (FTIR) spectroscopy were recorded utilizing a PerkinElmer Spectrum (PerkinElmer Inc., Waltham, Massachusetts, USA). A Jenway digital potentiometer (model 3330, UK), equipped with a glass electrode, was employed for accurate pH measurement. UV–visible absorption measurements were conducted using a Shimadzu double-beam spectrophotometer (model 1800, Tokyo, Japan) operated with UV Probe 2.43 software. The analysis employed matched quartz cuvettes, each possessing a 1 cm optical path length.

### Synthesis of O-CQDs

Carbon quantum dots were prepared using a direct carbonization approach. Precisely, 0.50 g each of urea, GA, and EDTA were meticulously combined and placed in a porcelain dish. After that, thermally treated on a hot plate at 270 °C for 30 min in air until charring occurred and a dark residue formed. The resulting material was finely ground, dispersed in 50.0 mL of double distilled water, and ultrasonicated for 15 min. The suspension was subsequently subjected to centrifugation at 6000 rpm for 45 min then filtered using a 0.22 μm syringe filter. The resultant brownish CQD filtrate was preserved at 4 °C to be used further as stock solution. The concentration of the stock (10.00 mg/mL) was determined after lyophilization of a known volume.

### Preparation of Working Solutions

A stock solution of TICA (100.00 µg/mL) was diluted in MeOH. The O-CQDS working solution is prepared by diluting 1.0 mL of stock in 100mL-volumetric flask (100 µg/mL) using distilled water.

### Quantum Yield Calculation

The quantum yield (QY) was calculated using the following comparative equation:

$$\:\boldsymbol{Q}={\boldsymbol{Q}}_{\boldsymbol{r}\boldsymbol{e}\boldsymbol{f}}\:\boldsymbol{X}\:\frac{\boldsymbol{I}}{{\boldsymbol{I}}_{\boldsymbol{r}\boldsymbol{e}\boldsymbol{f}}}\:\boldsymbol{X}\:\frac{{\boldsymbol{A}}_{\boldsymbol{r}\boldsymbol{e}\boldsymbol{f}}}{\boldsymbol{A}}\:\boldsymbol{X}\:\frac{{\boldsymbol{n}}^{2}}{{\boldsymbol{n}}_{\boldsymbol{r}\boldsymbol{e}\boldsymbol{f}}^{2}}$$

Where I and I_ref_ denote the integrated fluorescence intensities of the sample and reference, respectively; A and A_ref_ are their corresponding absorbance values; and n and n_ref_ are the refractive indices of the diluents used (distilled water = 1.33). The chemical used as reference fluorophore was quinine sulfate is dissolved in 0.10 M H₂SO₄, exhibiting a quantum yield of 0.54.

### Analytical Procedure for Assaying TICA

Different aliquots of TICA stock solution (100.00 µg/mL) were added to 10 mL volumetric flasks containing 50.0 µL of O-CQDs working solution (100 µg/mL). Each flask was completed to the mark with distilled water, and fluorescence intensity (FI) was subsequently determined at λ_em_ 417 nm after excitation at λ_ex_ 290 nm.

### Interference Study

To evaluate the selectivity of the developed fluorescent probe, an interference study was performed. A 10.0 mL volumetric flask was prepared containing 50.0 µL of the O-CQDs working solution and each potential interferent, adjusted to yield a final concentration of 50 mM. The tested interferents included clonidine hydrochloride, glucose, creatinine, calcium ions, magnesium ions, zinc ions, potassium ions, and ferrous ions. The solutions were diluted to the mark with distilled water, and the fluorescence intensity was recorded under the same optimized conditions as applied in the main assay.

### Assay of TICA in Pharmaceutical Preparation

Ten Brilique^®^ pills, each labelled to contain 90 mg of TICA, were weighed and finely ground. An equivalent amount of 10.00 mg of TICA powder was precisely measured and transferred into a 100 mL volumetric flask. Extraction was performed using 50.0 mL of methanol with the aid of ultrasonication, and the final volume was adjusted to the mark using the same solvent. The resulting solution (100.0 µg/mL) was filtered through a 0.45 μm syringe filter. Appropriate aliquots of the filtrate were subsequently added into a series of 10 mL volumetric flasks, each containing 50.0 µL of the working O-CQDs solution and diluted to volume with distilled water to obtain the desired concentrations. Afterwards, the same analytical procedure for assaying TICA was applied.

## Results and Discussion

### Characterization and Photoluminescent Features

**Fig. 1:**
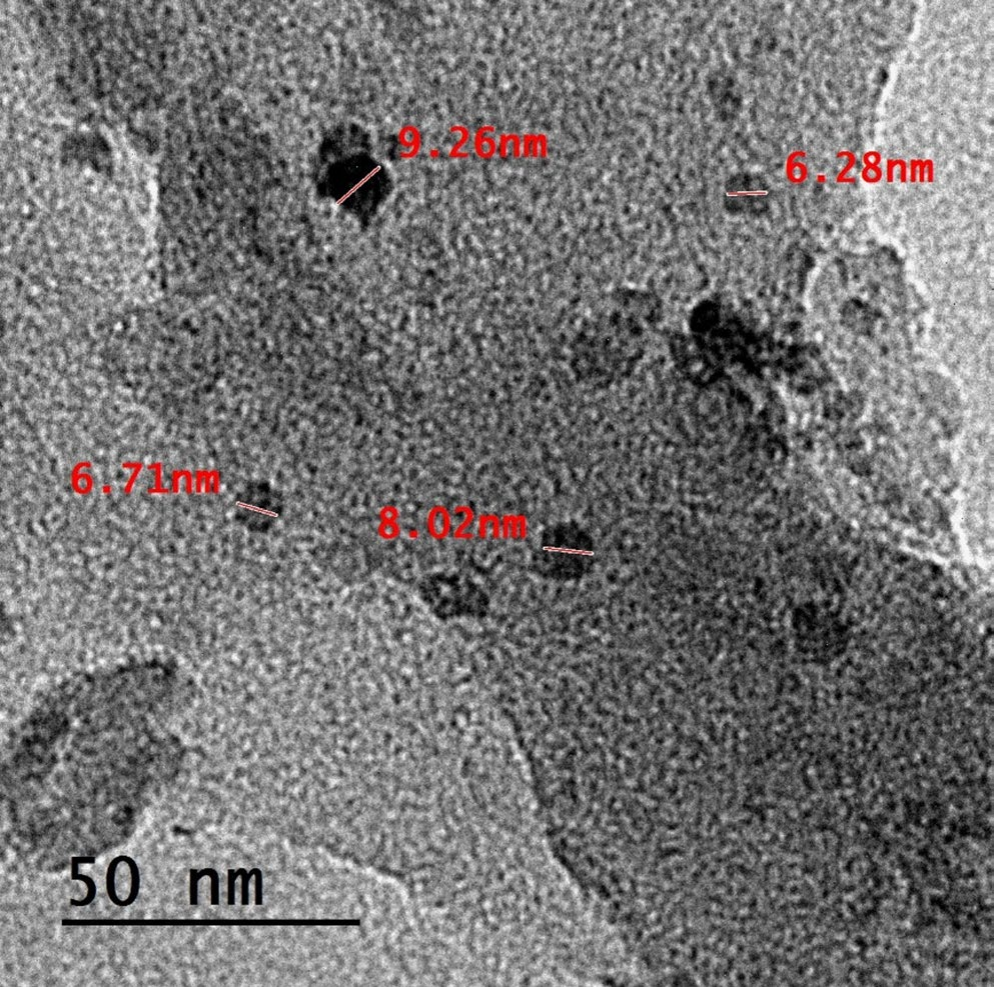
HRTEM image of carbon quantum dots showing different particles sizes

**Fig. 2:**
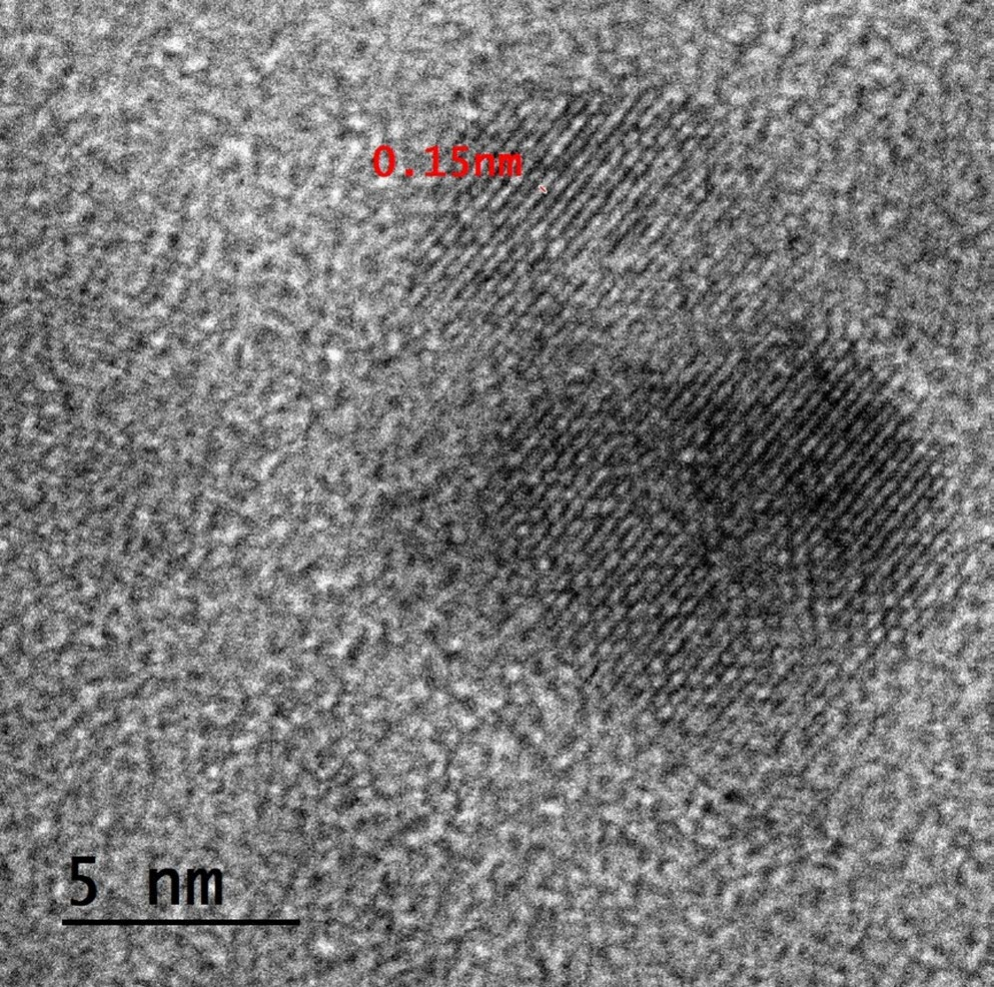
HRTEM images showing lattice fringes in the synthesized carbon quantum dots

**Fig. 3:**
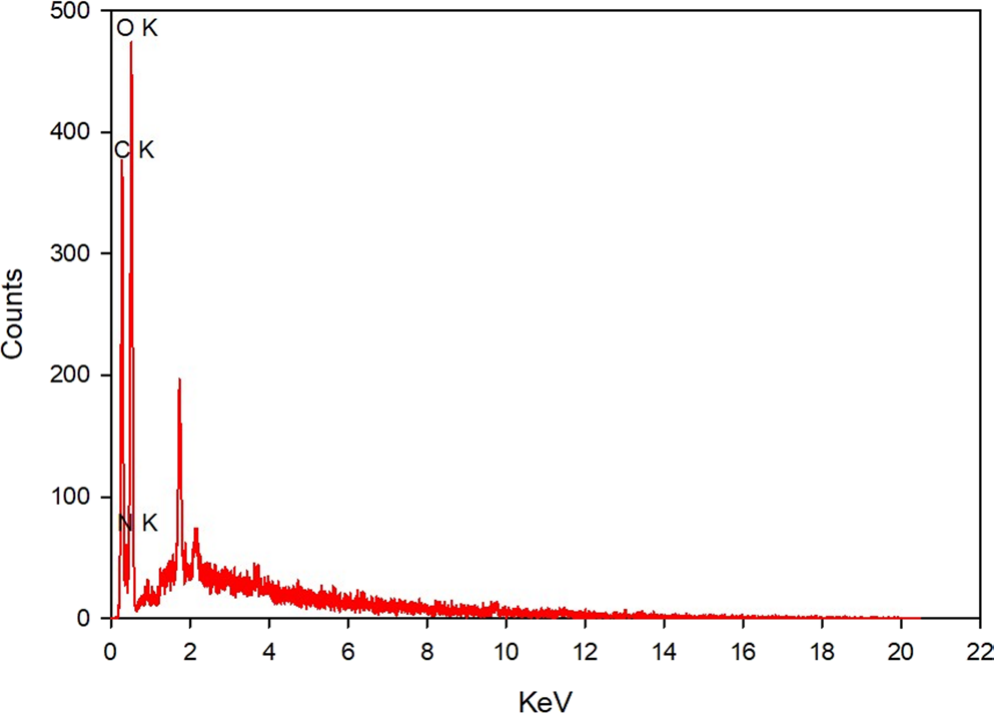
EDX analysis for carbon quantum dots conducted at an accelerating voltage of 20 kV, with a 200× magnification and an amplifier time of 0.24 µs

**Fig. 4:**
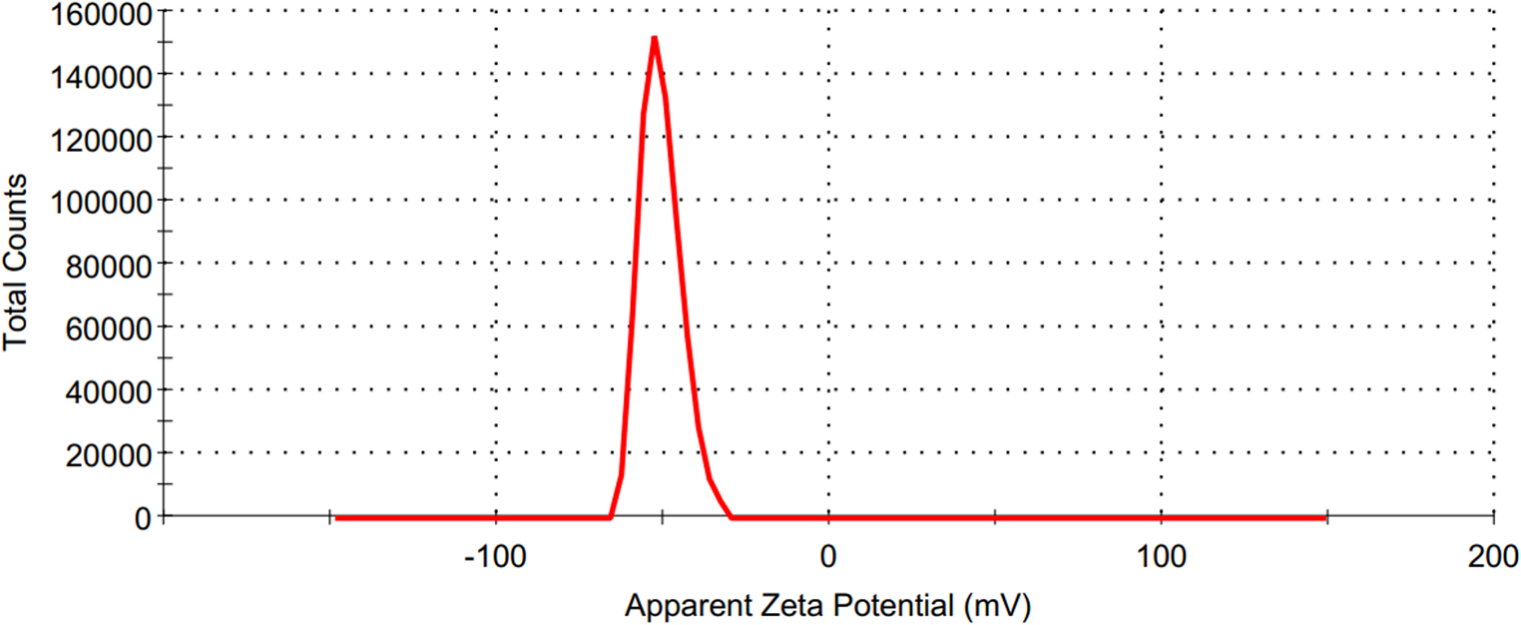
Zeta potential graph of the synthesized carbon quantum dots

**Fig. 5:**
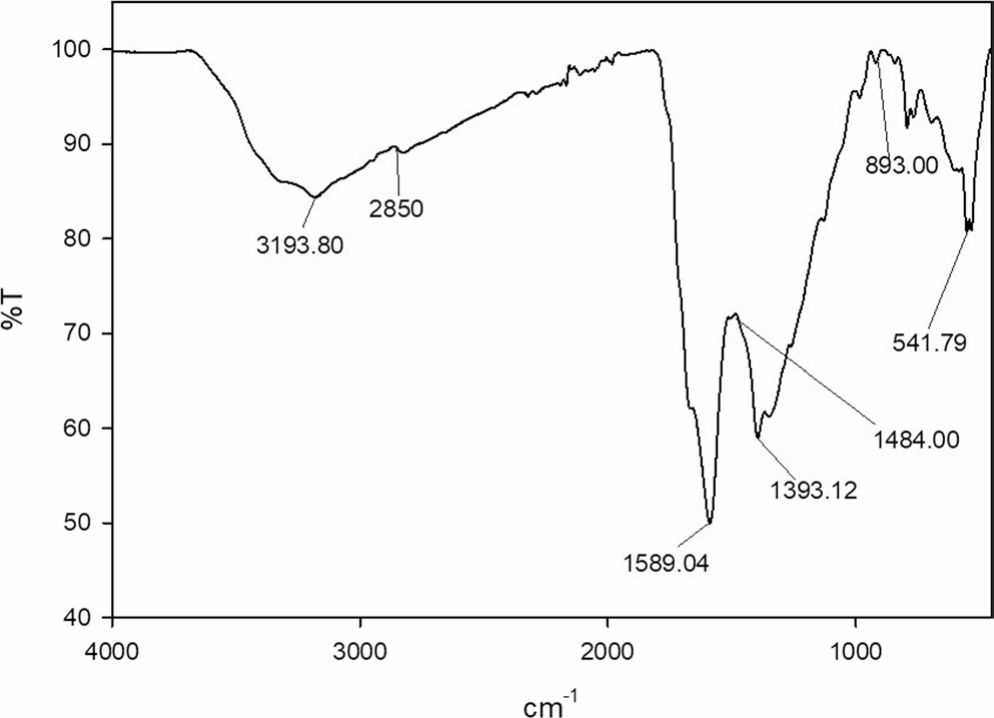
FTIR spectrum of the synthesized carbon quantum dots

The O-CQDs have bright blue fluorescence under long ultraviolet light (365 nm) as seen in Figure S1 (supplementary materials). HRTEM images (Fig. 1) reveal that the synthesized O-CQDs are uniformly dispersed and show a nearly spherical morphology with no evident aggregation. The measured particle sizes were consistently below 10 nm with average sizes 8.23 ± 0.84 nm, confirming the nanoscale nature of the synthesized dots. Clear lattice fringes with an interplanar spacing of 0.15 nm were distinctly seen (Fig. 2) aligning with the plane of graphitic carbon, confirming crystalline nature of the prepared O-CQDs. The elemental composition of the synthesized O-CQDs was examined using EDX analysis (Fig. 3), confirming the presence of nitrogen (N K) at 22.59%, carbon (C K) at 34.08%, and a notably high amount of oxygen (O K) at 43.33%. The prevalence of oxygen-containing functional groups suggests enhanced surface functionalization, which contributes not only to the hydrophilicity and fluorescence stability of the O-CQDs but also supports the development of a blue shift in the excitation spectrum [2]. The zeta potential and polydispersity index (PDI) of the prepared O-CQDs were evaluated, with values of − 50.70 mV (Fig. 4) and 0.35, sequentially. The significant negative zeta potential signifies strong electrostatic repulsion among particles, hence facilitating colloidal stability by inhibiting aggregation [13, 28]. The obtained PDI value indicates a rather narrow particle size dispersion, hence confirming the homogeneity of the synthesized O-CQDs. The FTIR spectrum of the synthesized O-CQDs displayed multiple unique absorption bands (Fig. 5), validating the effective integration of functional groups from the precursor materials. A broad band centered at 3400 cm⁻¹ was attributed to overlapping O–H and N–H stretching vibrations, signifying the presence of surface hydroxyl and residual amine functionalities. The absorption band at 2850 cm⁻¹ is indicative of symmetric and asymmetric C–H stretching vibrations of aliphatic carbon. A pronounced peak at approximately 1710 cm⁻¹ is ascribed to the C = O stretching vibration of carboxylic acid or amide groups. Furthermore, a band at 1589 cm⁻¹ was attributed to aromatic C = C stretching, signifying the preservation of aromatic compounds from gallic acid. Significant peak at 1393 cm⁻¹ is ascribed to the bending vibrations of CH_2_ and CH_3_ groups. Additionally, a band at 1250 cm⁻¹ corresponds to the C–O stretching of phenolic groups, whereas absorption at 1135 cm⁻¹ is ascribed to C–N and C–O–C stretching. Collectively, these spectral features validate the successful formation of O-CQDs bearing oxygen-rich functional groups, consistent with the chosen precursors and the intended synthesis route. After that, the QY of the developed O-CQDs was found to be 27.85% ± 0.89, demonstrating their strong fluorescence capability and suitability for sensitive analytical applications.

### Photostability Studies of O-CQDS

The photoluminescence stability of O-CQDs is significantly influenced by factors like pH, ionic strength, and light exposure duration. Consequently, these parameters must be meticulously examined before employing the prepared O-CQDs as a fluorescence-based probe for the detection of TICA. The FI of the O-CQDs was measured in pH ranges from 2.00 to 12.00 using BRB (Figure S2a). The results revealed a noticeable decrease in FI at acidic pH levels (2.00 to 5.00), followed by a gradual increase at pH 6.0. After that, FI demonstrated stability throughout the pH range extending from 7.00 to 12.00. This result validates the pH sensitivity of the synthesized O-CQDs and indicates their appropriateness for application in biological conditions. The observed fluorescence change based on pH can be ascribed to the protonation and deprotonation dynamics of functional groups on the O-CQD surface [29]. In acidic circumstances (pH < 5.00), surface functional groups are protonated, promoting non-covalent interactions and aggregation, which ultimately results in fluorescence quenching [30]. Conversely, at elevated pH levels (7.0–10.0), the deprotonation of oxygen-containing groups amplifies the negative surface charge, thereby reducing aggregation and augmenting fluorescence. Additionally, the FI of the synthesized O-CQDs was evaluated under varying ionic strength by introducing NaCl solutions at concentrations ranging from 40.00 to 280.00 mM (Figure S2b). The results indicated that the FI remained relatively stable across 50.00 to 200.00mM. The photostability of the prepared O-CQDs was assessed under prolonged UV exposure (254 nm). The FI remained nearly unchanged after 30 min of UV exposure (Figure S2c). The fluorescence has insignificant change upon its storage for 2 months in amber colored bottle at 4 °C. All these findings collectively demonstrate reliability of the synthesized O-CQDs. Their FI stability across a wide pH range, tolerance to moderate ionic strength, and resistance to light under prolonged UV exposure strongly support their suitability for use as effective fluorescent probes in analytical and biological applications.

### Comparison of the Performance of the Developed and the Reported Oxygen-Rich CQDs

The synthesized CQDs were benchmarked against other oxygen-rich CQDs reported in the literature. As summarized in Table 1, many of the published protocols suffer from significant limitations, such as the use of hazardous solvents and reagents such as sodium hydroxide, hydrochloric acid, and hydrogen peroxide [31–33]. Moreover, previously reported oxygen-rich CQDs generally displayed low QY values [32, 34]. In contrast, our method not only produced O-CQDs with markedly higher QY but also offered a rapid and straightforward synthesis route by classical pyrolysis of the bio-inspired substrates, making it time-efficient compared to other laborious and time-consuming approaches. These advantages highlight the superiority and practicality of the proposed method.

**Table 1:** Comparison of the performance of the developed carbon quantum dots with the previously reported oxygen rich carbon quantum dots

QY (%)	Precursor	Method and time consumption	Solvents used	Reference
9.91%	Peanut shell	Pyrolysis at 250 °C for 2 h.	water	[34]
25.7%	Chewing gum	Hydrothermal oxidation of discard chewing gum via H_2_O_2_ (3 wt %) at 180 °C for 8 h.	water	[32]
-	Chocolate	Chocolate was dispersed in 10 M NaOH solution and vigorously stirred then the mixture was reacted at 200 °C for 8 h using the solvothermal method and the product was neutralized using HCl.	water	[31]
-	Coffee bean shell	Coffee bean shells were dispersed in 4% aqueous NaOH for 1.5 h then the filtrate was acidified with 0.5 M HCl.	Methanol/water (1:1 v/v)	[33]
27.85%	Gallic acid, Urea, EDTA	Pyrolysis at 270 °C for 30 min.	water	Our method

### Investigation of Quenching Mechanisms

Several quenching mechanisms can be responsible for fluorescence quenching, including the inner filter effect (IFE), dynamic quenching, and static quenching. In dynamic quenching, collisions between the excited fluorophore and the quencher facilitate non-radiative deactivation, returning the fluorophore to its ground state without photon emission [35, 36]. In contrast, static quenching entails the creation of a non-luminescent ground-state complexes between the quencher and the fluorophore. Both dynamic and static quenching are temperature-dependent: in dynamic quenching, efficiency typically increases with temperature due to enhanced molecular collisions, while in static quenching, it tends to decrease as higher temperatures destabilize the ground-state complex. The IFE, however, is independent of temperature [37].

To investigate the quenching mechanism involved in the interaction between TICA and the synthesized O-CQDs, fluorescence quenching experiments were performed using different concentrations at two different temperatures. The procedure was performed in triplicate. The Stern–Volmer equation,

$$\:\frac{{\boldsymbol{F}}^{0}}{\boldsymbol{F}}=1+{\boldsymbol{K}}_{\boldsymbol{S}\boldsymbol{V}}\left[\boldsymbol{Q}\right]$$

was used to analyze the quenching behavior, where F^0^ and F denote the fluorescence intensity prior to and after the addition of TICA., respectively, [Q] is the TICA concentration, and K_SV_​ is the Stern–Volmer quenching constant. As shown in the corresponding Figure S3 and Table 2, the increase in K_SV_​ with rising temperature suggests a dynamic quenching mechanism.

**Table 2:** The quenching constant for the interaction between carbon quantum dots and ticagrelor, determined through fluorometric titration at two different temperatures

Temperature °K	K_SV_ (L. mole^− 1^) x 10^6^	Correlation coefficient
281	0.015	0.996
298	0.046	0.995

Moreover, the UV–Vis absorption profile of TICA showed considerable spectral overlap with the excitation wavelength of the synthesized O-CQDs (Figure S4), indicating that the IFE additionally assists to the observed fluorescence quenching. Therefore, the quenching of O-CQDs by TICA is likely governed by a combination of dynamic quenching and IFE.

### Optimization of Experimental Conditions

The optimal conditions for utilizing O-CQDs to achieve maximum FI were evaluated. The ideal O-CQDs concentration was identified to be 0.50 µg/mL at 290 nm, following an evaluation of concentrations ranging from 0.03 to 1.00 µg/mL, where maximum quenching efficiency was achieved as seen in Figure S5. The influence of TICA on the FI of O-CQDs was examined using BRB across pH ranging from 2.0 to 8.0. Interestingly, the quenching efficiency exhibited by TICA remained relatively constant throughout the evaluated pH values (Figure S6). The influence of incubation time was studied. The quenching of O-CQDs by TICA is stabilized very quickly. Equilibrium was reached within 1 min, as the fluorescence intensity remained constant over the 1–20 min interval (Figure S7).

### Determination of TICA

**Fig. 6:**
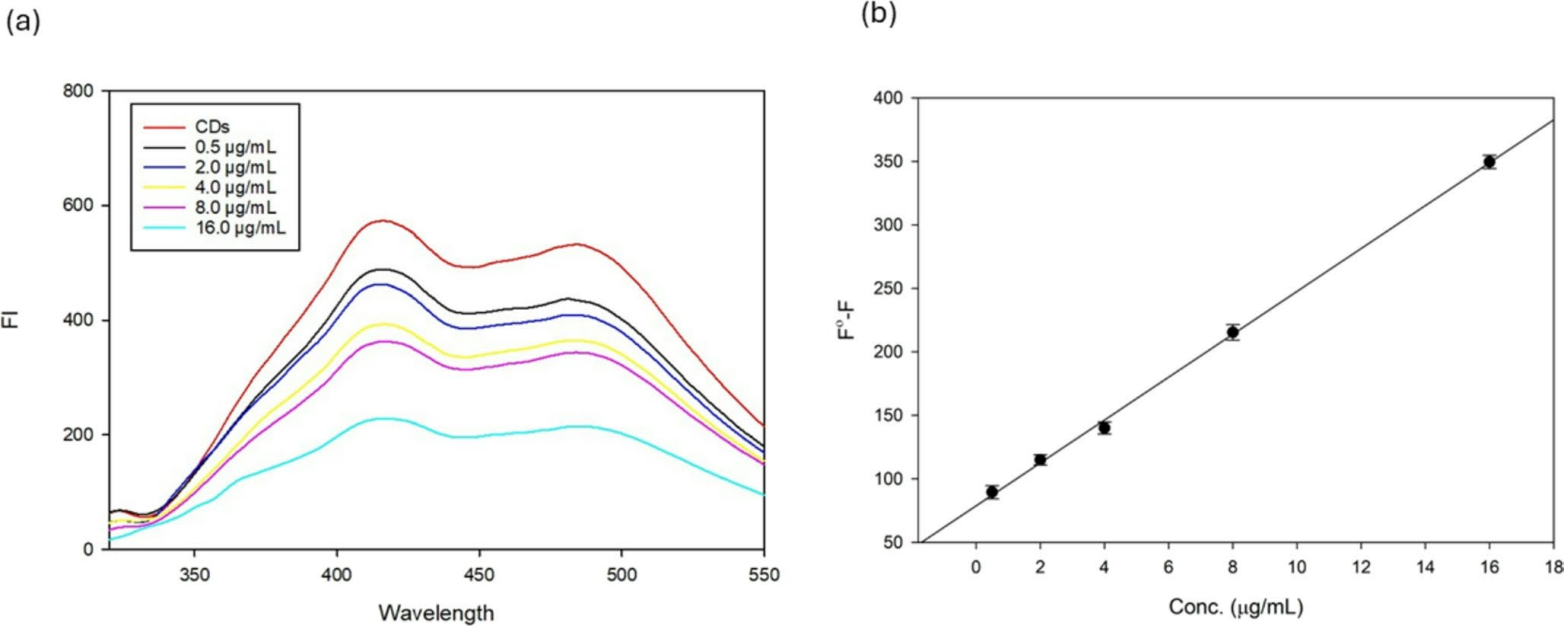
(**a**) Fluorescence spectra of carbon quantum dots under increasing ticagrelor concentrations, (**b**) calibration curve for the determination of ticagrelor using carbon quantum dots

The fluorescence intensity of the synthesized O-CQD-based probe quantitively diminished with the incremental addition of TICA, indicating a concentration-dependent quenching effect as shown in Fig. 6a. A linear relationship was noted between fluorescence quenching (F^0^–F) and TICA concentration across the 0.50–16.00 µg/mL range as shown in Fig. 6b. All measurements were performed in triplicate to ensure reliability. The methodological validation parameters according to ICH guidelines of the developed method are summarized in Table 3.

**Table 3:** Regression parameters for the established method to determine ticagrelor

Parameter	Value
Slope ± SD^a^	16.86 ± 0.33
Intercept	74.52 ± 2.06
Correlation coefficient	0.9994
Linearity range (µg/mL)	0.50–16.00
LOQ (µg/mL)^b^	0.50
LOD (µg/mL)^b^	0.21
Accuracy (mean ± SD)^c^	102.05 ± 1.57
Repeatability (%RSD)^d^	1.54
Reproducibility (%RSD)^d^	2.43
Robustness (%RSD)^e^	0.73

(a) Average of three determinations. (b) LOD was calculated from the following equation of 3.3(SD/Slope) where SD is standard deviation of residuals from the calibration curve. (c) results of three concentration 3,5,10 µg/mL of TICA. (d) Relative standard deviation of 3 concentrations 0.5,2,4 µg/mL TICA repeated three times on the same day, and 3 consecutive days calculated for repeatability and reproducibility, respectively. (e) Robustness was evaluated by deliberately varying the operating temperature (25 °C ± 2 °C) and the CQD solution volume (± 1 µL)

### Interference Study

**Fig. 7:**
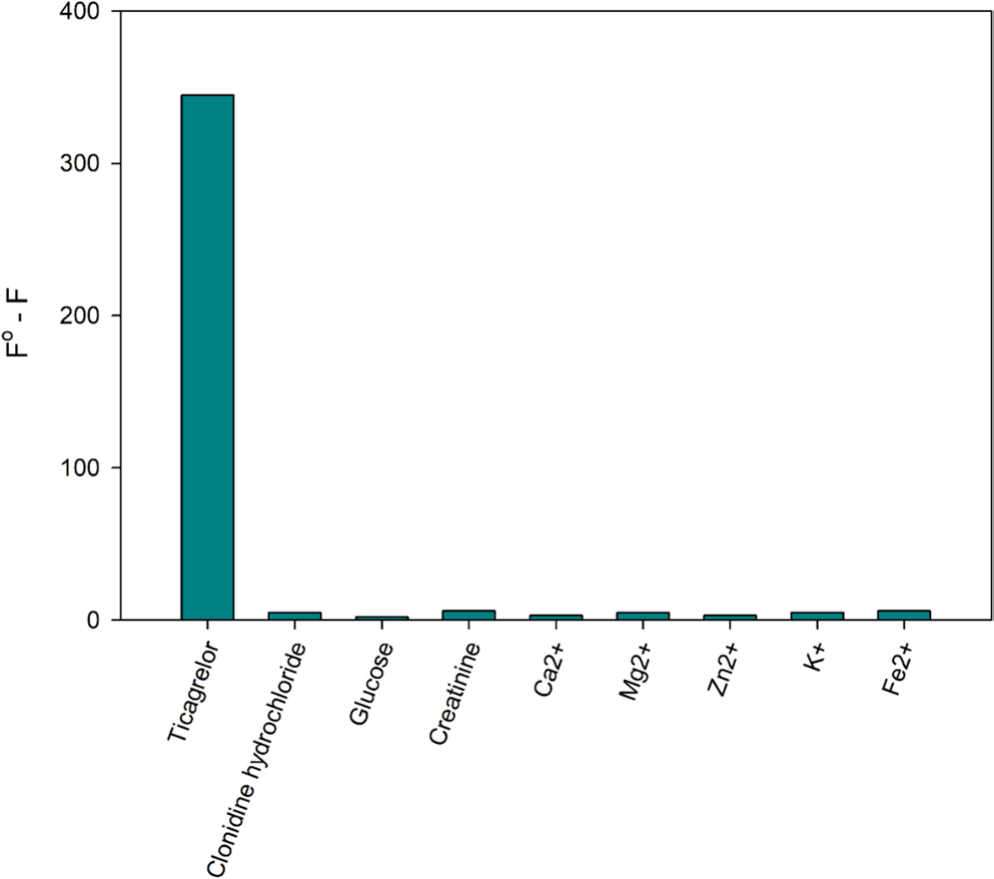
The effect of different interfering substances on the FI of the developed CQDs

The interference study demonstrated that the developed fluorescent probe maintained high selectivity. At a final concentration of 50 mM, none of the tested interferents including clonidine hydrochloride, glucose, creatinine, and biologically relevant metal ions (Ca²⁺, Mg²⁺, Zn²⁺, K⁺, Fe²⁺) produced any significant change in the fluorescence intensity of the O-CQDs under the optimized assay conditions as shown in Fig. 7. The recorded fluorescence values remained essentially constant compared to control samples containing O-CQDS only. These findings confirm that the observed quenching response is selective to TICA and not influenced by common interferants.

### Assay of TICA in its Pharmaceutical Formulation

The O-CQD-based fluorimetric system was successfully applied to determine TICA in its marketed dosage form, Brilique^®^ pills. The procedure was executed in triplicate, exhibiting high percentage recovery within the permitted limits and acceptable precision. A statistical comparison was carried out between the developed method and a published one [21] Student’s t-test and F-test were conducted as depicted in Table 4, indicating no significant difference at the 95% confidence level.

**Table 4:** Comparative statistical analysis of the proposed and reported methods for quantifying ticagrelor in pharmaceutical dosage forms

Brilique^®^*	Developed method	Published method**
Mean	98.32 ± 1.22	98.28 ± 0.92
n	3	6
Variance	1.49	0.85
Student’s t-test (2.37) at *p* = 0.05	0.06	
F-value (5.79) at *p* = 0.05	1.75	

*GTIN:06223003270537 SN:01578382807013

** Spectrophotometric method for the determination of Ticagrelor was established based on measuring the zero-order absorbance at 256.00 nm [21]

### Comparison of the Proposed Method with Other Reported Methods for TICA Determination

The developed- O-CQD-based fluorimetric probe for the analysis of TICA demonstrates several clear advantages over previously reported techniques (Table 5). Most notably, the proposed method achieves a low detection limit (0.21 µg/mL) that surpasses most reported spectrophotometric and HPLC approaches [16, 18, 20, 21]. Although electrochemical methods [25, 26] achieved slightly lower LOD values (0.15 µg/mL), their practical applicability is somewhat restricted. In particular, one of the reported approaches is prone to electrode fouling [26], necessitating frequent surface regeneration and thus limiting ease of routine use. Moreover, both methods rely on electrode modifications that add to experimental complexity, which compromise their practicality for routine quality control.

**Table 5:** Comparison of the proposed method with other reported methods for TICA determination

Method	Conditions	LOD (µg/mL)	LOQ (µg/mL)	Linearity (µg/mL)	Analysis time (min)	Reference
RP-HPLC	C18 column with mobile phase of acetonitrile: methanol (60:40%v/v) and UV detection at 254 nm	0.38	1.16	20–100	4.5	[16]
RP-HPLC	C18 column with mobile phase of acetonitrile: buffer solution (pH2.90) (75:25 &v/v) and UV detection at 256 nm	1.31	3.98	10–30	3.58	[18]
Spectrophotometry	Multi-variate regression at five wavelengths (253, 255, 257, 259, and 261 nm, with λ_max_ at 257 nm)	-	-	5–15	-	[20]
Spectrophotometry	Zero order (256.0 nm), and zero order AUC (251.2–263.0), first derivative order (261.2 nm), and first derivative under AUC (257.4–265.4 nm)	0.50	1.52	5–30	-	[21]
Square wave voltammetry	Carbon paste electrode modified with titanium dioxide nano particles	0.15	0.44	0.52–99.30	-	[25]
Differential pulse voltammetry	Au electrode in 0.05 M NaHCO_3_	0.15	0.50	0.16–5.2	-	[26]
Proposed method	Quenching the FI of the developed that synthesized via pyrolysis/30 min from GA, urea, and EDTA in distilled water via the dynamic and IFE quenching mechanisms.	0.21	0.50	0.50–16	< 1 min	

In contrast, the proposed fluorescent based probe is simple, rapid, and does not require complicated surface treatments or hazardous organic solvents. As far as we know, this is the first carbon dot–based fluorescent probe reported for the analysis of TICA, representing a new direction for nanomaterial-based pharmaceutical sensing.

Another advantage of the developed probe is its time efficiency. HPLC assays require multiple steps including equilibration, conditioning, separation, and column washing, resulting in analysis times exceeding several minutes per run. In contrast, fluorimetric platform achieves equilibrium and complete analysis in less than one minute, making it highly attractive for high-throughput routine use.

### Evaluation of the Ecological Friendliness and Practicality of the Proposed Method

**Fig. 8:**
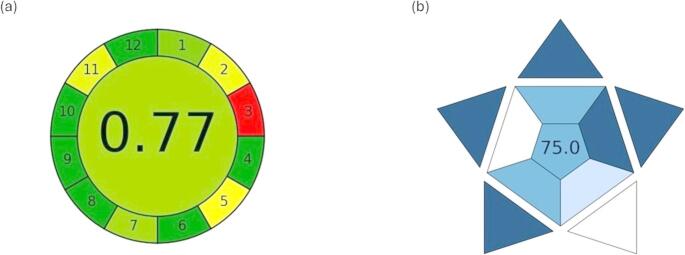
(**a**) AGREE pictogram representing the greenness profile of the developed method, (**b**) BAGI pictogram illustrating the practical applicability of the developed method

The developed fluorimetric method was assessed for its environmental sustainability complying with green chemistry standards in analytical methodology [38–40]. Green analytical chemistry (GAC) provides a framework to develop techniques that reduce hazardous waste, enhance user safety, and mitigate environmental impact, in addition to being cost-effective and efficient. The Analytical GREEnness (AGREE) metric was utilized to assess the ecological compatibility of the presented technique. This tool integrates all twelve GAC principles into a cohesive, user-centric assessment framework. The outcomes are depicted using a circular pictogram, in which each segment color indicates adherence to specific green chemistry principle [41]. The AGREE assessment validated that the developed method exhibits significant greenness with a score of 0.77 (Fig. 8a). This favorable greenness score can be ascribed to several factors. First, the method employs distilled water as the only solvent, thereby avoiding hazardous or toxic reagents. Second, the amount of solution consumed per measurement in fluorimetric analysis is minimal, further reducing waste. Finally, the assay requires no elaborate or multi-step sample preparation; the procedure simply involves the addition of a small aliquot of the CQD solution to the analyte, followed by direct fluorescence measurement. Together, these aspects underscore the method’s low environmental burden, operational simplicity, and compliance with green analytical chemistry principles.

In addition to its environmental benefits, the method’s overall applicability was further assessed using the Blue Applicability Grade Index (BAGI). This emerging tool complements green assessments by integrating practicality and real-world applicability—the “blue” dimension of White Analytical Chemistry [42]. The BAGI pictogram Fig. 8b yielded a score of 75.00, which clearly represents the method’s strengths across essential practical and analytical dimensions, reflecting its entire blueness. The high BAGI score reflects the method’s practicality, as it requires only a standard spectrofluorimeter, minimal operator training, and no complex sample preparation. Its rapid workflow, and minimal energy consumption make it well-suited for routine pharmaceutical analysis and other real-world applications.

The AGREE and BAGI evaluations together affirm that the suggested CQD-based fluorimetric method is environmentally friendly, widely applicable, and efficient for the routine analysis of TICA.

## Conclusion

This study presents the successful and sustainable synthesis of cost-effective O-CQDs via a straightforward pyrolysis method, achieving a quantum yield of 27.85 ± 0.89%. The resulting O-CQDs exhibited blue-shifted excitation maxima, attributed to the use of GA as a precursor rich in oxygen-containing functional groups. These O-CQDs demonstrated excellent fluorescence characteristics, rapid analytical response, and notable stability, enabling their effective application as a fluorescent probe for the quantification of TICA in both bulk powder and pharmaceutical formulation. The method was rigorously evaluated and validated, showing high accuracy, precision, selectivity, and robustness. Furthermore, greenness and operational practicality were confirmed using the AGREE and BAGI assessment tools, supporting the method’s alignment with green and applicable analytical standards. Overall, the findings underscore the potential of oxygen-rich, blue-shifted O-CQDs as a versatile and reliable platform for advanced pharmaceutical and biomedical analysis.

## Supplementary Information

Below is the link to the electronic supplementary material.

Supplementary Material 1 (DOCX 348 KB)

## Data Availability

Information can be acquired by reaching out to the corresponding author with a formal request.
