# Supplementary material for: Blue Shifted Carbon Dots-Based Fluorescent Probe for Determination of Ticagrelor: A Dual Method Evaluation Via AGREE and BAGI
Source: J Fluoresc. 2025 Nov 17;36(2):1285–95. doi: 10.1007/s10895-025-04618-y (PMC12979407; doi:10.1007/s10895-025-04618-y)
Supplement: Supplementary file 1 — Supplementary Material 1 (DOCX 348 KB) [file 10895_2025_4618_MOESM1_ESM.docx]

**Blue Shifted Carbon Dots-Based Fluorescent Probe for Determination of Ticagrelor: A Dual Method Evaluation via AGREE and BAGI**

Abdelrahman M. Allam*, Shereen A. Boltia, Azza Aziz M. Moustafa, Sally S. El-Mosallamy

Pharmaceutical Analytical Chemistry Department, Faculty of Pharmacy, Cairo University, Kasr El-Aini St., P.O. Box 11562, Cairo, Egypt.

* Corresponding author, email: [abdelrahman.magdy@pharma.cu.edu.eg](mailto:abdelrahman.magdy@pharma.cu.edu.eg)


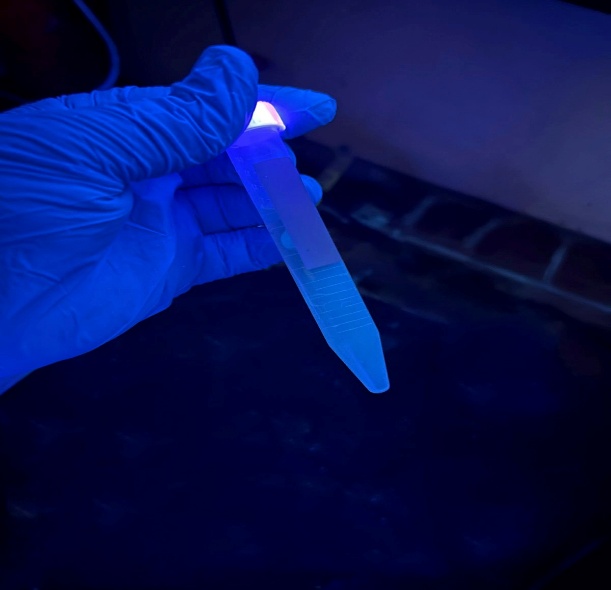


Figure S1: The bright blue fluorescence emitted by the synthesized CQDs under 365 nm ultraviolet light


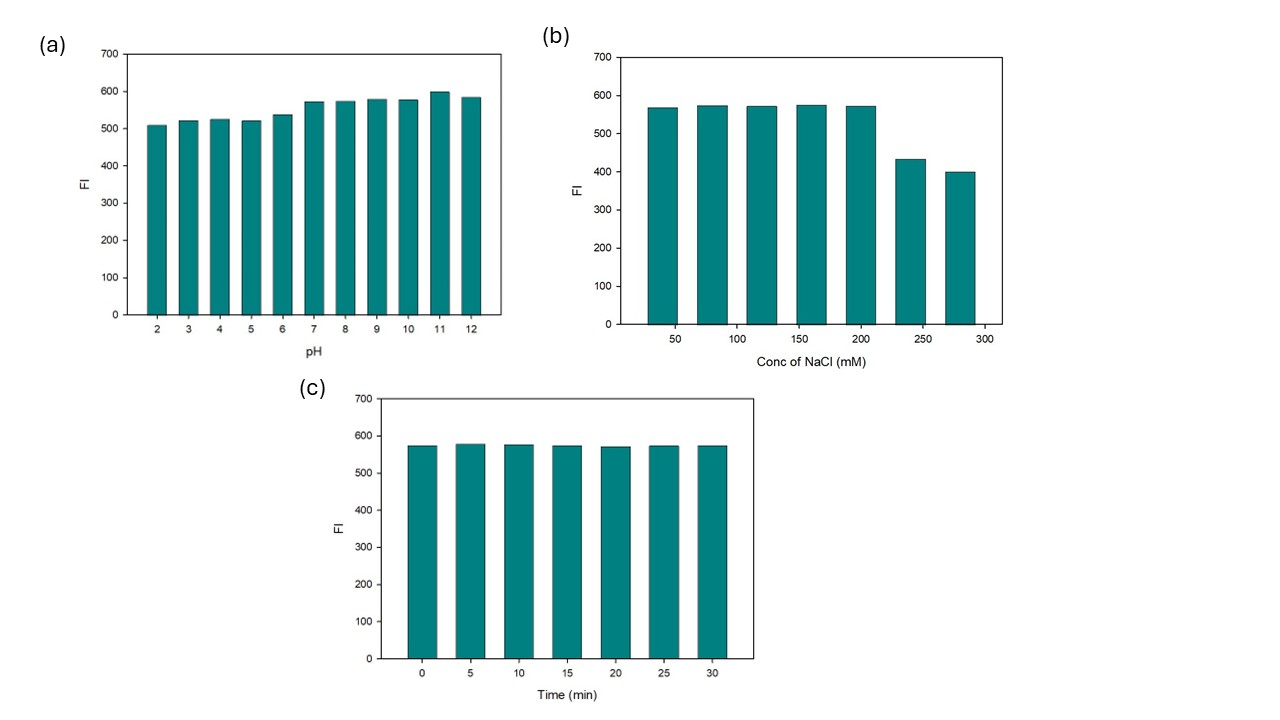


Figure S2: The effect of (a) pH, (b) ionic strength, (c) light exposure on the FI of synthesized CQDs


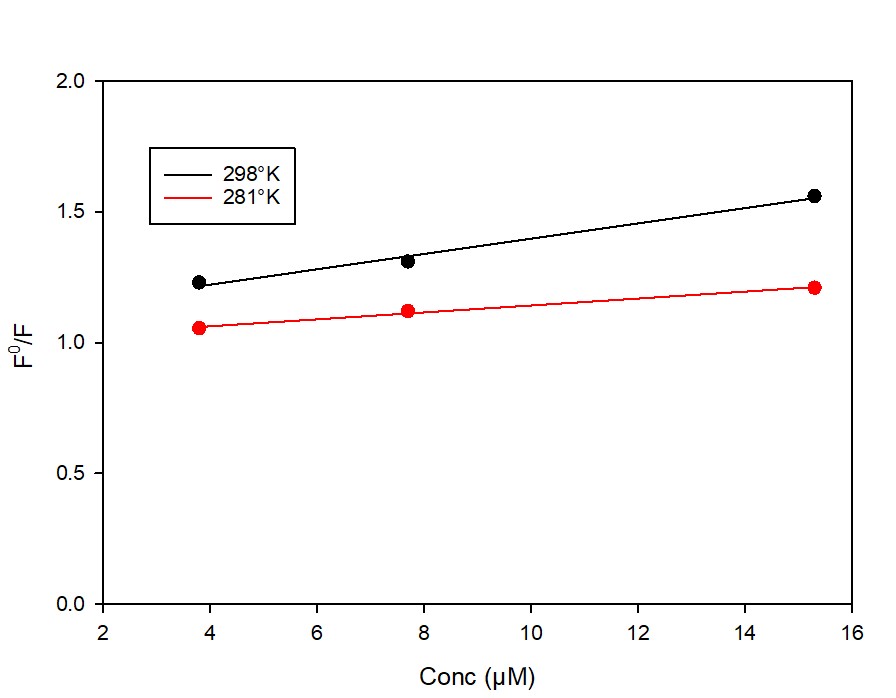


Figure S3: Stern–Volmer plots illustrating the interaction between ticagrelor and carbon quantum dots at two distinct temperatures


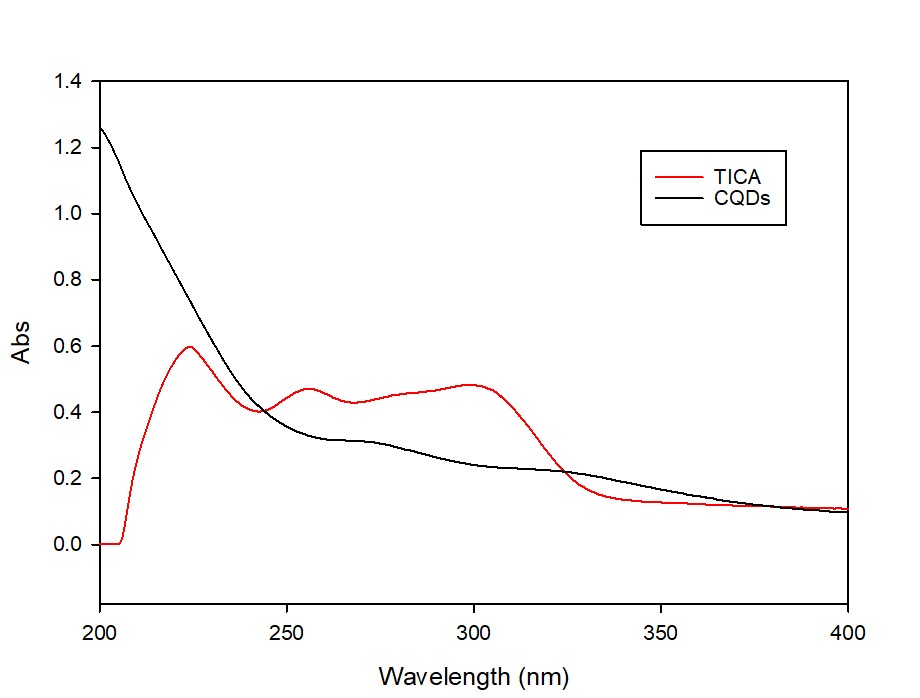


Figure S4: UV-vis spectra of the prepared CQDs and TICA


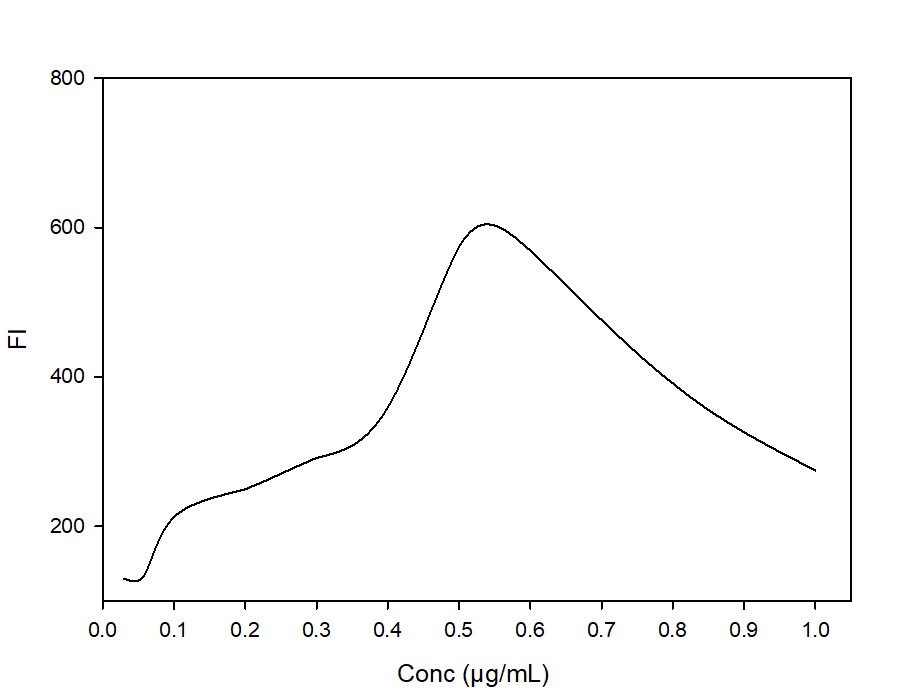


Figure S5: Effect of varying CQD concentrations on FI


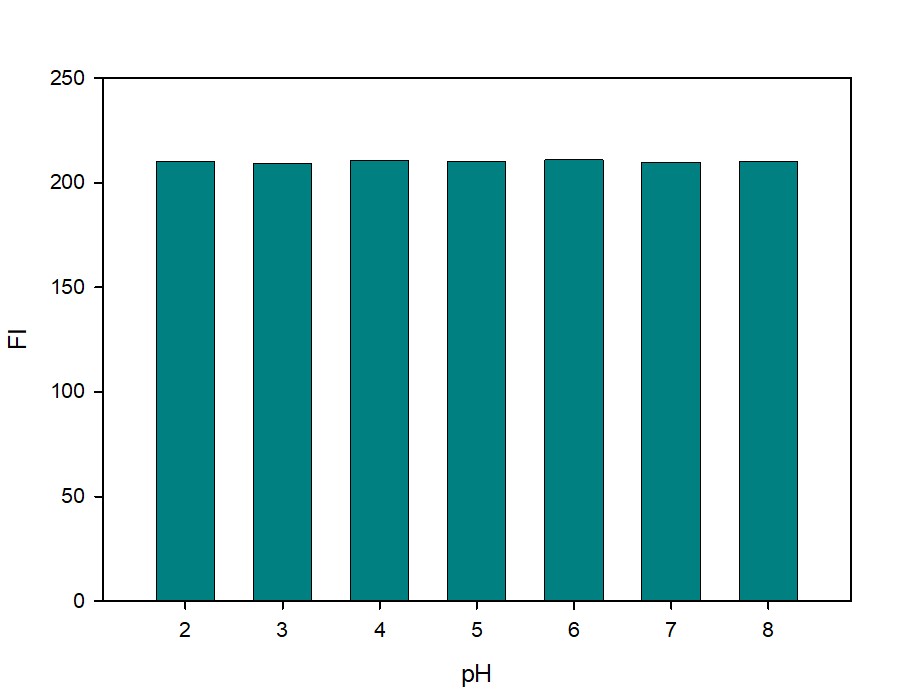


Figure S6: Effect of pH on TICA (8.00 µg/mL) fluorescence quenching efficiency


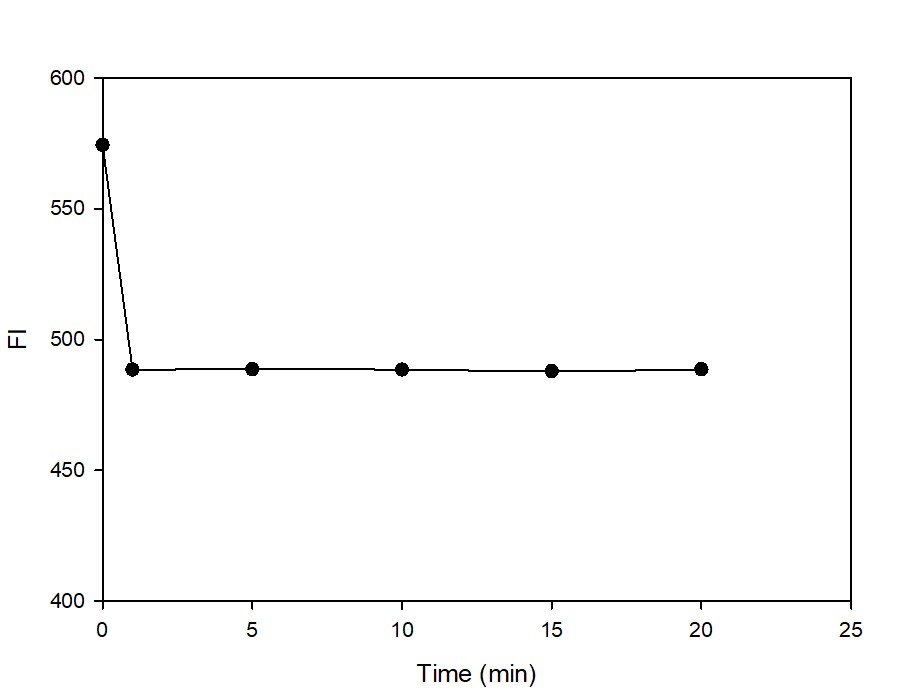


FigureS7: Effect of incubation time on the interaction between TICA and CQDs
